# Female homicides in Brazil before and during the COVID-19 pandemic: an interrupted time-series analysis

**DOI:** 10.1186/s12889-025-24814-6

**Published:** 2025-10-24

**Authors:** Karen Raquel Ferreira do Nascimento, Rafael Tavares Jomar, Glauber Weder dos Santos Silva, Eder Samuel Oliveira Dantas, Karina Cardoso Meira

**Affiliations:** 1 Graduate Program in Demography, Federal University of Rio Grande do Norte, Natal, Rio Grande do Norte Brazil; 2 Assistance Coordination, National Cancer Institute, Rio de Janeiro, Rio de Janeiro, Brazil; 3 Public Health Department of the State of Rio Grande do Norte, Natal, Rio Grande do Norte Brazil; 4 Onofre Lopes University Hospital, Federal University of Rio Grande do Norte, Natal, Rio Grande do Norte Brazil; 5 Department of Pharmaceutical Sciences, Federal University of São Paulo, São Paulo, Brazil

**Keywords:** Homicide, Femicide, Brazil, COVID-19, COVID-19 pandemic, Interrupted time series analysis, Ecological studies

## Abstract

**Background:**

The COVID-19 pandemic has influenced violence against women worldwide, but its impact on female homicides in low- and middle-income countries remains poorly understood. Brazil, which records some of the highest female homicide rates globally, provides a critical setting to examine this association. This study assessed the temporal association between the pandemic and monthly female homicide rates in Brazil from January 2017 to December 2022.

**Methods:**

We applied an interrupted time series (ITS) design with quasi-Poisson regression to estimate changes in homicide levels and trends after the pandemic onset, adjusting for serial autocorrelation and seasonality. Pre-pandemic trend linearity was tested, and sensitivity and placebo analyses were performed. To address underreporting and deaths classified as undetermined intent, homicide counts were corrected for misclassification.

**Results:**

From 2017 to 2022, 23,727 female homicides were recorded, corresponding to an adjusted mortality rate of 5.09 per 100,000 women, a 16.0% increase after correction. Rates were highest in the North and Northeast. Domestic homicides exceeded those in public spaces (1.50 vs. 1.37 per 100,000 women), and firearms were the predominant method. The Northeast showed a significant level change with an abrupt increase (RR = 1.236; *p* = 0.002), followed by a progressive decline (RR = 0.9923; *p* < 0.001). In other regions, across age groups, and in blunt-related cases, no significant level change occurred (*p* > 0.05), although downward trends emerged during the pandemic (*p* < 0.05).

**Conclusions:**

Findings warrant cautious interpretation due to ITS constraints, sensitivity to the observation window, and omitted variables. Nonetheless, persistently high female homicide rates in Brazil, particularly in the Northeast, highlight the need to strengthen mortality surveillance, improve misclassification corrections, and adopt region-specific prevention strategies, including firearm control, protective services, and targeted social policies.

**Supplementary Information:**

The online version contains supplementary material available at 10.1186/s12889-025-24814-6.

## Background

Latin America and the Caribbean (LAC) have historically been marked by conquest, instability, and violence, a situation exacerbated by their position along international trafficking routes for drugs, goods, and people, which renders women and children especially vulnerable to violent acts [1–3]. In 2022, despite comprising only 8% of the global population, this region accounted for 29% of all homicides worldwide [4]. Brazil alone was responsible for approximately 10.04% of global homicides in 2021 (46,000 vs. 458,000) [5].

The progressive increase in lethal violence among men in LAC often translates into higher female homicide rates, as societies with high male homicide levels tend to nurture sociocultural norms, such as machismo, the cult of virility, and conflict resolution through private violence, that also foster gender-based violence [6–12]. Empirical studies linking social disorganization, socioeconomic deprivation, and criminality demonstrate that as these structural determinants intensify, homicide rates for both sexes rise [13–16].

With the advent of the COVID-19 pandemic, scholars forecasted an increase in domestic violence, female homicides, and femicides based on past evidence of increased violence following disasters, conflicts, and economic crises [17–19]. Criminological theory suggests that mobility restrictions exacerbate stress and negative emotions, which, alongside higher alcohol and drug use, elevate the risk of violent behavior [19, 20].

Indeed, several studies reported a rise in domestic violence complaints after COVID-19 containment measures were introduced in Turkey, France, and Brazil [19]. Paradoxically, female homicide records declined during the same period [21–25]. Similarly, in Chile, Mexico, and Peru, domestic violence and female homicide indicators followed a “U-shaped” trajectory between 2020 and 2021—initially dropping at the pandemic’s onset before rebounding to or beyond pre-pandemic levels [21–25].

Brazil continues to report a high burden of domestic violence and persistently elevated rates of female homicide and femicide [15, 16]. In this setting, the non-pharmaceutical interventions adopted to curb the spread of SARS-CoV-2, in particular social-distancing mandates, interacted with pre-existing structural fragilities. The collapse of an already strained health-care system and an inadequate social safety net deepened institutional vulnerabilities, thereby magnifying the pandemic’s adverse effects. Simultaneously, enforced isolation, the scaling-back of victim support services, worsening socioeconomic conditions, and chronic under-funding of the violence-prevention network intensified women’s exposure to lethal violence [26–30].

Given this context, this study aims to examine the temporal association between the COVID-19 pandemic period and monthly female homicide rates in Brazil from January 2017 through December 2022, employing an interrupted time-series analysis to characterize changes in level and trend.

## Methods

### Study design and location

This ecological study employs an interrupted time series (ITS) design, in accordance with the Guidelines for Accurate and Transparent Health Estimates Reporting (GATHER) [31]. We analyzed monthly homicide rates among women residing in Brazil, aged 10–14 years and 80 years or older, over a 72-month period: 38 months before (January 2017–February 2020) and 34 months after (March 2020–December 2022) the first confirmed case of COVID-19 (February 26, 2020). March 2020 was selected as the intervention point because, in the first weeks of that month, all states implemented social distancing measures and mobility restrictions, providing a clear and uniform temporal marker.

Given the potential for unmeasured time-varying confounding, as well as the inability to fully verify key ITS assumptions, such as the stability of the pre-intervention trend, the absence of concurrent events, the estimated changes in level and trend are interpreted as temporal associations rather than causal effects [32].

### Data source

Female homicide records were obtained from the Mortality Information System (SIM), using external cause mortality data provided by the Department of Informatics of the Brazilian Unified Health System (DATASUS). We included deaths classified under the 10th Revision of the International Classification of Diseases (ICD-10), specifically:

- X85–Y09: Homicides (assaults);
- X93–X95: Firearm-related homicides;
- X99–Y00: Blunt objects; and.
- Y35: Legal intervention deaths, these refer to actions carried out by State agents (such as police officers or military personnel) in the course of their duties that result in injury or death. These codes are applied when the intent is legal or authorized, but the outcome is injurious or fatal. Scholars argue that Y35 may conceal extrajudicial killings and hinder accountability. Its use can obscure patterns of lethal state violence, especially against marginalized groups.

Additionally, we included deaths classified as events of undetermined intent (EUI), covering the following:

- Y10-Y34: Undetermined intent;
- Y22-Y24: Firearm-related injuries of undetermined intent;
- Y28-Y29: Blunt objects of undetermined intent.

The study variables included region of residence (North, Northeast, Southeast, South, Central-West), age group (10 to 14, 15 to 19, 20 to 39, 40 to 59, and 60 + years), method of perpetration (firearm vs. blunt object), and location of occurrence (household vs. public space), which are among the most prevalent homicide characteristics in Brazil [1, 15, 16, 33–35].

The population data were obtained from the Brazilian Institute of Geography and Statistics (IBGE), based on the 2024 edition of the Population Projection of Brazil and its Federative Units by sex and age for the period from 2000 to 2070.

Changes in the proportion of events of undetermined intent (EUI) may introduce bias into homicide estimates. In Brazil, between January 2017 and December 2022, we observed marked differences in the quality of information on deaths from external causes, with a high proportion of EUI particularly in the Southeast and Northeast regions and among homicides involving blunt objects (Supplementary Material 1) [24, 25]. This heterogeneity indicates that an unstratified correction, that is, one not accounting for region, place of occurrence, and method could distort or overstate adjustments in specific groups, thereby increasing information bias [33, 36].

To address this limitation, we implemented a proportional redistribution of EUI by year, month, and age group, based on the relative share of homicides among the total deaths from accidental, self-inflicted, assault-related, and legal intervention causes (ICD-10 code Y35), stratified by region, place of occurrence, and method. This procedure preserves the demographic and temporal structure of the data, preventing corrections from being disproportionately concentrated in specific periods or age strata [33, 34, 36].

### Correction of deaths process

The correction process followed three steps [33]:

1. Proportion of total homicides: We calculated the proportion of homicides relative to the total number of external cause deaths: including accidental (W00 to 59); assault (X85 to Y09); self-harm (X60 to X84), and legal interventions (Y35), disaggregated by month/year and age group.
2. Redistribution of EUI cases: The proportions obtained in Step 1 were applied to the total number of deaths classified as EUI (Y10–Y34), disaggregated by month/year and age group.
3. Correction of female homicide underreporting due to misclassification: The estimated values from Step 2 were added to the originally recorded X85–Y09 homicide cases in SIM/DATASUS, disaggregated by month/year and age group.

The correction procedure was applied to total homicide counts by locality, stratified by method of perpetration and location of occurrence. The entire process was independently conducted by two researchers and cross-verified by a third researcher (Supplementary Material 2).

### Data analysis

We calculated annual and monthly female homicide rates stratified by region, age group, location of occurrence, and method of perpetration. These rates were standardized via the direct method, adopting the World Health Organization (WHO) standard population as a reference [37].

To assess temporal changes, we estimated monthly mean standardized homicide rates, dividing the data into two periods: the 38 months preceding the first confirmed COVID-19 case (January 2017 to February 2020) and the 34 months following the initial diagnosis (March 2020 to December 2022).

To examine temporal trends in standardized monthly female homicide rates stratified by region, age group, and location of occurrence, we constructed line graphs and applied LOESS (locally estimated scatterplot smoothing) to smooth the trends.

### Interrupted time series analysis

We adjusted for female homicide mortality rates via a segmented regression model for each of the variables under study (region, age group, place of occurrence and method). The response variable was the number of female homicides, whereas the independent variable encompassed all months from 2017 to 2022 (ranging from 1 to 72 months). To assess level changes, we coded the pre-pandemic period as 0 and assigned a value of 1 to the months following the onset of the pandemic [38–40].

Given the characteristics of the response variable (a count variable), the Poisson regression model may be employed initially [41]. However, the dispersion test proposed by Cameron and Trivedi (1990) revealed the presence of overdispersion [42], the variance of the data exceeds the mean, thereby rendering the Poisson model inadequate for these data [38–41].

Thus, a quasi-Poisson regression model was employed to account for the overdispersion commonly observed in count data, allowing the variance to be proportional, but not necessarily equal to the mean. In this model, the variance is specified as Var(Y)= ϕ. µ. Where ϕ is a dispersion parameter estimated from the data, providing greater flexibility than the standard Poisson model [41]. The level and trend change model examined both the abrupt shift in homicide rates and the gradual trend change in monthly female homicide rates following the onset of the COVID-19 pandemic [38–41].

To evaluate the plausibility of the linearity assumption in the pre-intervention segment [39, 42], we fitted three quasi-Poisson regression models including linear (t), quadratic (t²) and cubic (t³) time terms to each series. Time was centred at the midpoint of the study period to mitigate multicollinearity among higher-order polynomials. Model fit was compared using the quasi-F statistic derived from deviance analysis, calculated as (ΔDeviance/Δdf)/ϕ, where ϕ denotes the dispersion parameter. We also computed the Quasi-Akaike Information Criterion (QAIC), defined as QAIC = D + 2 ϕ k, with D representing the quasi-deviance and k the number of model parameters [43].

We conducted separate interrupted time series (ITS) analyses for Brazil as a whole, the five macroregions, selected age groups (10 to14, 15 to 19, 20to 39, 40 to 59 and ≥ 60 years), methods of perpetration (firearms vs. blunt objects), and occurrence settings (domestic vs. public space). In each model, the unit of observation was the month *t* within stratum *s*; the same generic specification was estimated independently for every s in Eq. (1):

1 $$\begin{array}{c} {Y}_{s,t}{\sim}\mathrm{Quasi-Poission}(\mu_{s,t})\\ \:{log}\left({\mu\:}_{s,t}\right)=\:{\beta\:}_{0}+\:{\beta\:}_{1}T\:+\:{\beta\:}_{2}{X}_{t}\\+\:{\beta\:}_{3}\left(T\:-\:{T}_{0}\right){X}_{t}+offset\:\left({log}\left({Pop}_{s,t}\right)\right)\end{array}$$

where 𝑌_s_,_𝑡_ represents the monthly count of female homicides in stratum *s* at month *t* and $\:{\mu\:}_{s,t}$,its expected mean. The covariate $\:T$ counts months since January 2017 ($\:T$ =72), while $\:{X}_{t}$ equals 0 before the pandemic and 1 from March 2020 onward ($\:T$ =39). The parameter $\:{\beta\:}_{0}$ denotes the baseline level at $\:T=0$; $\:{\beta\:}_{1}$ captures the pre-intervention trend in homicide rates over time; $\:{\beta\:}_{2}$ represents the immediate level change at pandemic onset; and ​$\:{\beta\:}_{3}$ quantifies the slope change following that intervention. Finally, the term $\:offset\:\left({log}\left({Pop}_{s,t}\right)\right)$ ensures that the model estimates rates (female homicides). By including the $\:\left({log}\left({Pop}_{s,t}\right)\right)\:$ as an offset, we effectively normalize the outcome by the size of the female population in each stratum, so that exponentiated coefficients correspond to rate ratios (RR) [38, 39].

In constructing the models, we evaluated the presence of seasonality. There are some strategies to control these patterns, including indicator variables for calendar months or quarters, seasonal differencing, splines, and Fourier terms (pairs of sine and cosine functions with a 12-month periodicity). We used Fourier terms, specifically two harmonic pairs corresponding to the annual cycle, because they provide a continuous, parsimonious representation of seasonal fluctuations. By using only four parameters, this approach preserves the integrity of the long-term trend and simplifies inference on intervention effects. Moreover, Fourier terms maintain the series’ stationarity without requiring differencing and eliminate the need for knot selection, thereby enhancing both computational efficiency and model interpretability [38–40, 44, 45].

The analyse is represented by the following Eq. (2) [37–39, 45].

2 $$\begin{aligned} {log}\left({\mu\:}_{t}\right)=&{\beta\:}_{0}+\:{\beta\:}_{1}T\:+\:{\beta\:}_{2}{X}_{t}+\:{\beta\:}_{3}\left(T\:-\:{T}_{0}\right){X}_{t}\:\\&+\:offset\:\left({log}\left({Pop}_{t}\right)\right)+{\gamma\:}_{1}{sin}\frac{2\pi\:X\left(t\right)}{L}\:\\&+{\gamma\:}_{2}{cos}\frac{2\pi\:X\left(t\right)}{L}+{\gamma\:}_{3}{sin}\frac{4\pi\:X\left(t\right)}{L}\\&+{\gamma\:}_{4}{cos}\frac{4\pi\:X\left(t\right)}{L}\end{aligned}$$

where the Fourier terms are $\:{\gamma\:}_{1}\:sin\:\frac{2\:\pi\:X\left(t\right)\:}{L}$, $\:{\gamma\:}_{2}\:cos$ $\:\frac{2\:\pi\:X\left(t\right)\:}{L}$, $\:{\gamma\:}_{3}\:sin$ $\:\frac{4\:\pi\:X\left(t\right)\:}{L}$, and $\:{\gamma\:}_{4}\:cos$ $\:\frac{4\:\pi\:X\left(t\right)\:}{L}$. *L* represents the seasonality period (12 months); $\:{\gamma\:}_{1}\:e\:{\gamma\:}_{2}$ are coefficients associated with the fundamental frequency $\:\frac{2\:\pi\:X\left(t\right)\:}{L}$. $\:{\gamma\:}_{1}$ is the amplitude of the sinusoidal component of the fundamental frequency, $\:{\gamma\:}_{2}$ is the amplitude of the cosine component of the fundamental frequency, $\:{\gamma\:}_{3}\:e\:{\gamma\:}_{4}$ are coefficients associated with the first harmonic $\:\frac{4\:\pi\:X\left(t\right)\:}{L}{,\:\:\gamma\:}_{3}$ is the amplitude of the sinusoidal component of the first harmonic, and $\:{\gamma\:}_{4}$ is the amplitude of the cosine component of the first harmonic [38–40, 44, 45].

Initial exploratory models were estimated under the assumption that the residuals were independent and identically distributed, and final models incorporated autoregressive terms whose order p. The presence of serial autocorrelation in residuals was assessed using the Durbin–Watson test alongside inspection of the ACF and PACF plots [45–47]. Significant PACF peaks dictated the inclusion of one or more AR(p) terms, yielding the final autoregressive model specification [45–47].

The model selection prioritized parsimony and goodness-of-fit criteria, including deviance and residual deviance measures [38, 39]. Once the optimal quasi-Poisson model was identified, relative risk (RR) estimates were obtained by exponentiating the estimated coefficients (β) [38, 39].

### Sensitivity analysis

This sensitivity analysis was conducted to assess the robustness of the ITS method in detecting changes in the level and trend of female homicide rates during the COVID-19 pandemic. Following the guideline that interrupted time series (ITS) models should include at least 12 observations before and 12 after the intervention to produce stable estimates of level and slope parameters [32, 38–40, 43, 47, 48], we restricted the analysis to the period from January 2019 to February 2021. This interval provided 14 pre-pandemic and 12 post-pandemic data points, ensuring adequate statistical power while limiting the temporal horizon to a period in which large, uncorrelated fluctuations in homicide rates were less likely. By re-estimating the quasi-Poisson segmented regression within this narrower window, we evaluated whether the coefficients for the immediate level change (β₂) and slope change (β₃) maintained direction and magnitude comparable to those obtained from the full 2017 to2022 series [32, 38–40].

### Placebo analysis

To assess the temporal specificity of the association between the COVID-19 pandemic and female homicide rates, and thereby mitigate the risk of attributing to the pandemic a structural break that could have arisen from other causes, we conducted a placebo (falsification) analysis by relocating the intervention point to September 2019, approximately six months before the first confirmed COVID-19 case in Brazil (26 February 2020). This date was chosen because it precedes any plausible pandemic-related behavioral, economic, or institutional changes, yet is sufficiently close to the actual intervention point to preserve comparability in seasonal patterns and underlying macrostructural conditions.

The segmented quasi-Poisson regression model used in the main analysis was re-estimated without any modification. The intervention indicator was defined as X_t_ = 0 through August 2019 and X_t_ = 1 from September 2019 onward, enabling estimation of the level effect (β₂) and the slope effect (β₃). Under the null hypothesis of this falsification test, if the pandemic-attributed impact is genuine, the estimated coefficients β₂ and β₃ ​ in the placebo model should yield non-significant p-values (*p* > 0.05), indicating that they are statistically indistinguishable from zero. In contrast, the same parameters at the true intervention point (March 2020) are expected to yield statistically significant p-values (*p* < 0.05). Observing large p-values for β₂ and β₃​ in the placebo model, alongside statistically significant results in the main analysis, increases confidence that the detected change is temporally aligned with the pandemic rather than reflecting unrelated fluctuations. Nonetheless, this approach does not fully rule out alternative explanations such as unmeasured concurrent events, residual confounding, or model misspecification [32, 38–40].

All analyses were conducted at a significance level of *p* < 0.05 via R statistical software (version 4.4.1), incorporating the following packages: lmtest, Epi, tsModel, gls, glmm, splines, vcd, nlme, ggplot2, AER, and tidyverse.

## Results

In Brazil, the Ministry of Health recorded 23,727 homicides of women between January 2017 and December 2022, corresponding to a rate of 4.28 homicides per 100,000 women. After mortality records were adjusted for misclassification errors, we identified a 16.02% increase in mortality rates, reaching 5.09 homicides per 100,000 women. This increase is attributed to the high proportion of cases initially classified as EUI within the information system, particularly in the Southeast and Northeast Regions of the country (Table 1).

**Table 1:** Female homicide rates (2017–2022) by correction Stages, Brazil

Locality	UR^a^	EIU^b^	RAIQ^c^	Percentage change (%)^d^
North	6.77	1.08	7.21	6.63
Northeast	5.88	3.62	6.71	14.14
Southeast	2.72	5.29	3.50	28.90
South	4.04	1.36	4.27	5.70
Midwest	5.20	1.81	5.63	8.42
Brazil	4.28	3.66	5.09	16.02
Place of occurrence	UR^a^	EIU^b^	RAIQ^c^	Percentage change (%)
At home	1.32	0.66	1.50	12.14
Public space	1.l21	0.18	1.37	11.19
Methods	UR^a^	EIU^b^	RAIQ^c^	Percentage change (%)
Firearm	2.21	0.07	2.28	2.87
Blunt objects	1.40	0.46	1.84	30.93

^a^Uncorrected standardized rates

^b^Standardized Rates of events with undetermined intent

^c^Standardized rates after correction of death records

^d^ Percentage change (comparison between standardized rates after correction and uncorrected standardized rates)

Source: Mortality Information System (SIM/SUS) | National Bureau of Statistics (IBGE)

The highest percentage increases in mortality rates following data correction were observed in the Southeast (29.80%) and Northeast (14.14%) regions (Table 1). The substantial increase in Southeast is due primarily to the high proportion of deaths initially classified as EUI. In this region, the number of deaths recorded as EUI was 1.95 times greater than those officially coded as homicides (5.29 vs.2.72 per 100,000 women) (Table 1).

The North and Northeast regions of Brazil exhibited the highest age-standardized female homicide rates, at 1.42 and 1.32 times higher, respectively, than the national average of 5.09 deaths per 100,000 women. Conversely, the Southeast and South were the only regions with rates below the national average (Table 1).

The homicide rate for women killed inside their residences was 9% higher than that for those killed in public spaces (1.50 vs. 1.37 homicides per 100,000 women). When methods of homicide were analyzed, firearm-related deaths were more incident than those caused by blunt objects (2.28 vs. 1.84 homicides per 100,000 women) (Table 1).

**Fig. 1:**
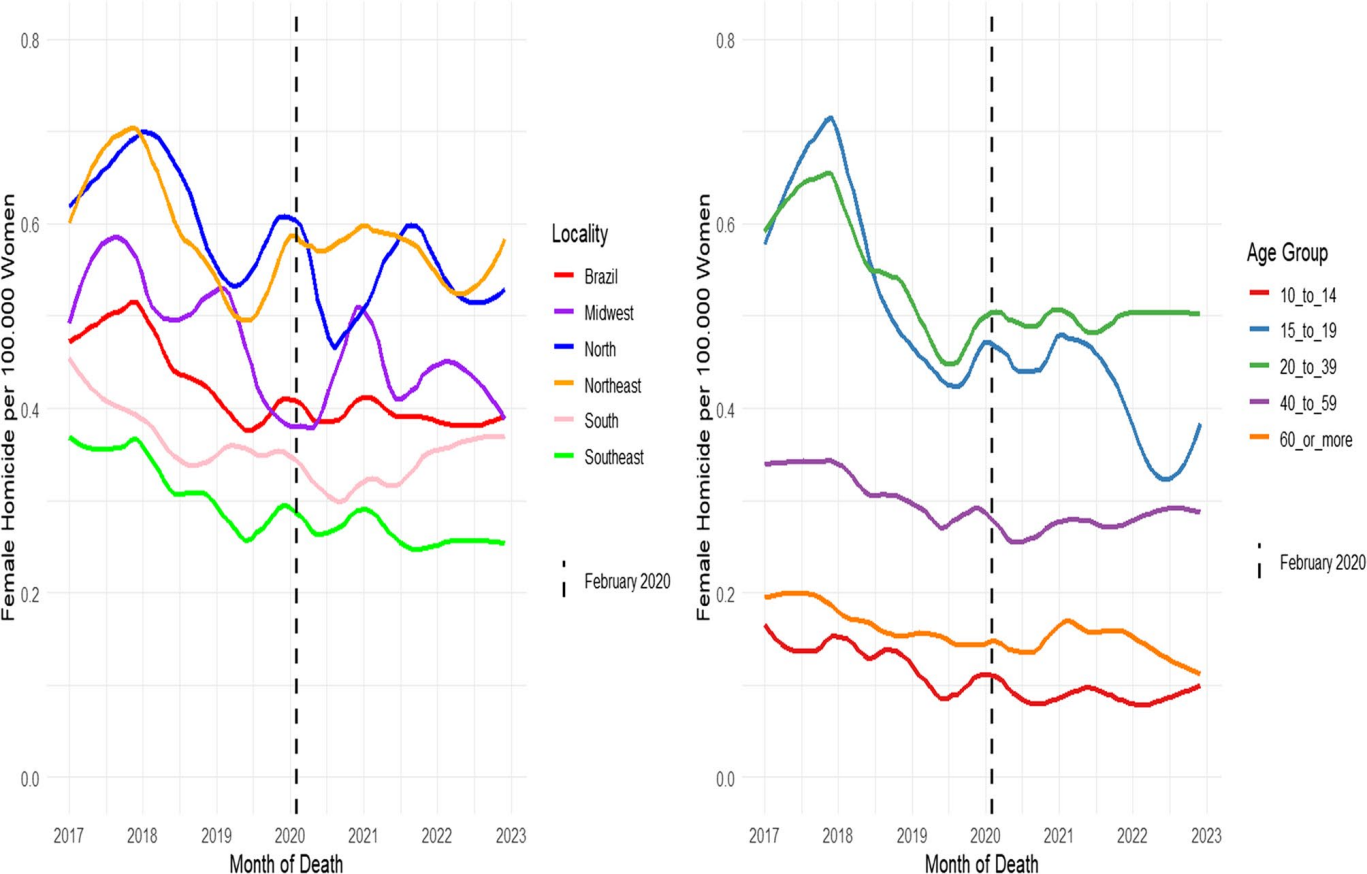
Monthly female homicide rates per 100,000 women in Brazil by region and age group, January 2017 to December 2022

**Fig. 2:**
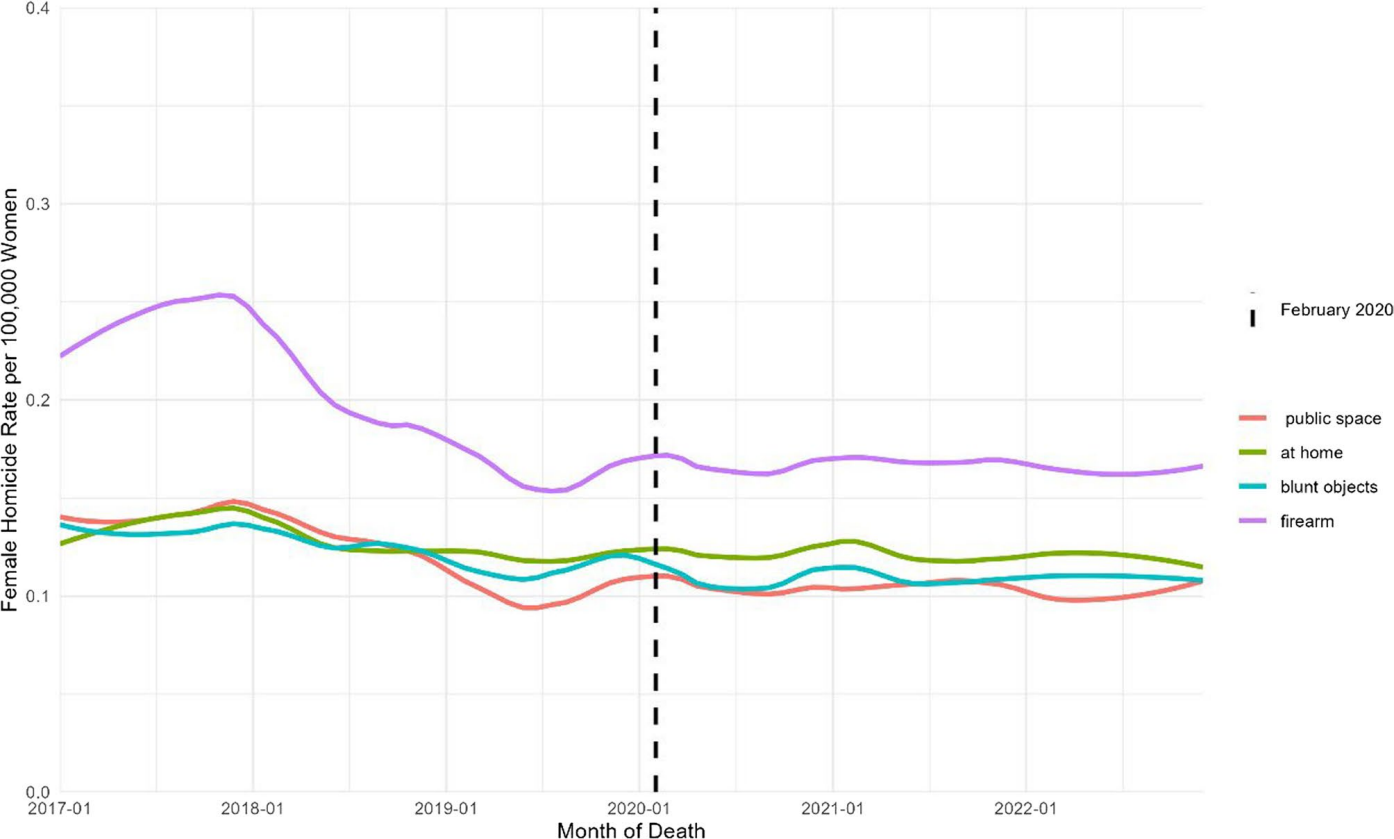
Monthly Female Homicides Rates per 100,000 Women by place of occurrence and method, January 2017 to December 2022

Applying LOESS smoothing to the monthly female-homicide rate series (Figs. 1 and 2) reveals a sustained downward trajectory that begins in 2017 and deepens through December 2019. Introducing the intervention point in March 2020 produces no discernible inflection; from that date onward, the curves stabilize at slightly lower levels, interrupted only by low-amplitude fluctuations. Regional heterogeneity nevertheless persists: the North and Northeast retain the highest coefficients, albeit converging, whereas the Southeast consistently registers the lowest. Age-stratified analysis indicates a steeper decline among women aged 15 to 19 years and 20 to 39 years up to 2019; post-intervention, a plateau emerges with a modest uptick restricted to adolescents during 2021 to 2022 (Fig. 1).

Disaggregation by mechanism and setting (Fig. 2) confirms the predominance of firearm-related homicides, whose rate falls until 2019 and thereafter stabilizes at approximately 0.18 per 100,000 women. Homicides involving blunt objects, as well as those occurring within the home or in public spaces, remain between 0.10 and 0.14 per 100,000 women, and exhibit no abrupt slope change after the pandemic onset, maintaining the stabilization pattern through December 2022 across all three categories examined (Fig. 2).

To evaluate the plausibility of the linearity assumption in the pre-intervention segment, we fitted quasi-Poisson regression models with linear (t), quadratic (t²), and cubic (t³) terms for each series. Model adequacy was assessed sequentially, linear vs. quadratic, and quadratic vs. cubic, using the quasi-F test (*p* < 0.05 indicating significance) and the change in Quasi-Akaike Information Criterion (ΔQAIC ≥ 2 indicating substantive improvement). In most strata, including all age groups and the Southeast, South, and Center-West regions, adding polynomial terms did not significantly reduce quasi-deviance (quasi-F *p* ≥ 0.05) or improve QAIC (ΔQAIC < 2), supporting the linear specification. Conversely, in the Northeast (quasi-F *p* < 0.001; ΔQAIC ≥ 2), at the national level (*p* = 0.001), among firearm-related victims (*p* = 0.026), and in public-space events (*p* = 0.015), the cubic model provided a statistically significant improvement, indicating that the pre-intervention trend deviated from linearity (Supplementary Material 3).

Given these findings, segmented regression models comparing the post-pandemic (March 2020 to December 2022) and pre-pandemic (January 2017 to February 2020) periods were fitted for each stratum, with results summarized in Table 2.

**Table 2:** Effect of the COVID-19 pandemic on female homicide rates per 100,000 women through ITS analysis series estimated by the quasi-Poisson regression model (January 2017 to December 2022)

Variable	Categories	Interpretation	β	SE	RR^a^	CI95%^b^	*p*-value
Locality	North*						
	β_1_	Stationary trend	−0.0047	0.0028	0.995	0.989–1.001	0.0981
	β_2_	Not detected	−0.1138	0.0877	0.892	0.751–1.059	0.1990
	β_3_	Not detected	−0.0027	0.0021	0.997	0.993–1.001	0.2080
	Northeast**						
	β_1_	Downward trend	−0.0084	0.0019	0.992	0.988–0.995	0.0001
	β_2_	Abrupt increase	0.2122	0.0543	1.236	1.111–1.375	0.0002
	β_3_	Progressive decrease	−0.0077	0.0014	0.992	0.989–0.994	0.0000
	Southeast***						
	β_1_	Downward trend	−0.0089	0.0019	0.991	0.987–0.995	0.0001
	β_2_	Not detected	0.0776	0.0594	1.081	0.961–1.214	0.1960
	β_3_	Progressive decrease	−0.0071	0.0015	0.993	0.990–0.995	0.0000
	South**						
	β_1_	Downward trend	−0.0073	0.0023	0.993	0.988–0.997	0.0037
	β_2_	Not detected	−0.0475	0.0815	0.954	0.8128–1.1186	0.5610
	β_3_	Not detected	−0.0008	0.0020	0.999	0.9952–1.0031	0.6760
	Midwest*						
	β_1_	Downward trend	−0.0090	0.0031	0.991	0.985–0.997	0.0060
	β_2_	Not detected	0.0584	0.0975	1.060	0.87–1.283	0.5509
	β_3_	Progressive decrease	−0.0056	0.0023	0.994	0.989–0.998	0.0188
	Brazil***						
	β_1_	Downward trend	−0.0080	0.0012	0.992	0.989–0.994	0.0000
	β_2_	Not detected	0.0622	0.0387	1.064	0.986–1.147	0.1122
	β_3_	Progressive decrease	−0.0051	0.0009	0.995	0.993–0.996	0.0000
Age group (years)	10 to 14***						
	β_1_	Downward trend	−0.0108	0.0046	0.989	0.980–0.998	0.0233
	β_2_	Not detected	−0.0692	0.1485	0.933	0.697–1.248	0.6426
	β_3_	Not detected	−0.0063	0.0036	0.994	0.987–1.001	0.0865
	15 to 19 ***						
	β_1_	Downward trend	−0.0129	0.0028	0.987	0.982–0.993	0.0000
	β_2_	Not detected	0.1686	0.0923	1.183	0.987–1.418	0.0723
	β_3_	Progressive decrease	−0.0124	0.0023	0.987	0.983–0.992	0.0000
	20 to 39***						
	β_1_	Downward trend	−0.0082	0.0014	0.992	0.989–0.994	0.0000
	β_2_	Not detected	0.0768	0.0481	1.079	0.982–1.186	0.1152
	β_3_	Progressive decrease	−0.0043	0.0012	0.995	0.993–0.998	0.0005
	40 to 59***						
	β_1_	Stationary trend	−0.0038	0.0020	0.996	0.992-1.000	0.0673
	β_2_	Not detected	−0.0555	0.0587	0.946	0.843–1.06	0.3481
	β_3_	Not detected	−0.0011	0.0015	0.998	0.996–1.001	0.4650
	60 or more***						
	β_1_	Downward trend	−0.0081	0.0029	0.992	0.986–0.998	0.0081
	β_2_	Not detected	0.1511	0.0990	1.163	0.957–1.412	0.1319
	β_3_	Progressive decrease	−0.0075	0.0024	0.992	0.987–0.997	0.0033
Methods	Firearm ***						
	β_1_	Downward trend	−0.0128	0.0017	0.987	0.984–0.991	0.0000
	β_2_	Abrupt increase	0.1308	0.0587	1.139	1.015–1.278	0.0295
	β_3_	Progressive decrease	−0.0087	0.0014	0.991	0.988–0.994	0.0000
	Blunt objects**						
	β_1_	Stationary trend	0.0007	0.0014	1.001	0.9980–1.003	0.5983
	β_2_	Not detected	−0.0677	0.0522	0.934	0.843–1.035	0.2000
	β_3_	Not detected	0.0013	0.0013	1.001	0.998–1.004	0.3329
Place of occurrence	At home***						
	β_1_	Downward trend	−0.004	0.0015	0.995	0.992–0.998	0.0030
	β_2_	Not detected	0.0777	0.0525	1.080	0.975–1.198	0.1437
	β_3_	Progressive decrease	−0.0037	0.0013	0.996	0.993–0.998	0.0057
	Public Space**						
	β_1_	Downward trend	−0.0113	0.0020	0.988	0.985–0.993	0.0000
	β_2_	Not detected	0.1202	0.0648	1.127	0.993–1.280	0.0682
	β_3_	Progressive decrease	−0.0081	0.0016	0.991	0.988–0.995	0.0000

^a^β (Beta)estimated coefficient from the quasi-Poisson regression model using a log link function, β_1__-_pre-intervention trend, β_2-_ represents the immediate level change at pandemic onset; β_3-_ quantifies the slope change following that intervention

^b^SE (Standard Error): standard error of the estimated β, indicating the precision of the coefficient estimate

^c^RR (Rate Ratio): exponential of β (exp(β))

^d^95% CI: 95% confidence interval for the RR, calculated as exp(β ± 1.96 × SE)

*model without seasonality and without lags in the autoregressive process

** model with seasonality and with 1 lag in the autoregressive process (AR(1))

*** model without seasonality and with 1 lag in the autoregressive process (AR(1))

Source: Mortality Information System (SIM/SUS) | National Bureau of Statistics (IBGE)

In the ITS analysis, the national aggregate exhibited a significant pre-intervention downward trend (β₁ = −0.0080; RR = 0.992; *p* < 0.001), with no abrupt level change (β₂ non-significant) but a progressive post-intervention decline (β₃ = −0.0051; RR = 0.995; *p* < 0.001). In the Northeast, a pre-existing decline (β₁ = −0.0084; RR = 0.992; *p* < 0.001) was followed by an immediate increase in level (β₂ = 0.2122; RR = 1.236; *p* < 0.001) and a subsequent accelerated reduction (β₃ = −0.0077; RR = 0.992; *p* < 0.001). The Southeast, South, and Center-West sustained declining trends both before and during the pandemic, with no evidence of level changes, whereas the North remained stable (Table 2).

By age group, the steepest pre-intervention decline occurred among individuals aged 15–19 years (β₁ = −0.0129; RR = 0.987; *p* < 0.001), with continued reduction post-intervention (β₃ = −0.0124; RR = 0.987; *p* < 0.001). Children aged 10–14 years also experienced a pre-intervention decline (β₁ = −0.0108; RR = 0.989; *p* = 0.023) but showed no subsequent changes. Young adults (20–39 years) and older adults (≥ 60 years) maintained pre-intervention decreases, with further reductions observed throughout the pandemic, while those aged 40–59 years exhibited no significant effects (Table 2).

Firearm-related homicides declined prior to the pandemic (β₁ = −0.0128; RR = 0.987; *p* < 0.001), increased abruptly after the intervention (β₂ = 0.1308; RR = 1.139; *p* = 0.030), and subsequently resumed a progressive decline (β₃ = −0.0087; RR = 0.991; *p* < 0.001). Homicides involving blunt objects displayed a stationary pre-pandemic trend, with no statistically significant changes in level or slope following the intervention. In contrast, homicides occurring in residences or public spaces maintained pre-pandemic downward trends without abrupt level shifts but experienced continued progressive reductions during the pandemic (Table 2).

### Sensitivity analysis

Application of this sensitivity framework revealed a marked attenuation in the statistical evidence for pandemic-related shifts in female homicide trends. In the full 2017 to 2022 models, most strata, encompassing multiple regions, age groups, methods, and places of occurrence, exhibited statistically significant pre-pandemic declines (β₁), abrupt level changes (β₂), and/or post-intervention trend shifts (β₃). However, when the series was truncated to January 2019 to February 2021, most of these associations lost statistical significance (Table 3).

**Table 3:** Sensitivity analysis analysis of the interrupted time series model, comparing the beginning of the pandemic in March 2020, with the beginning in September 2019

BP (Jan 2017 to Feb 2020) and DP (Marc 2020 to Dec 2022)						Sensitivity analysis- BP (Jan 2019 to Fev 2020) DP (March 2020 to Feb 2021)				
Variable	Categories	Interpretation	β	SE	*p*-value	Categories	Interpretation	β	SE	*p*-value
Locality	North*					North*				
	β_1_	Stationary trend	−0.0047	0.0028	0.995	β_1_	Stationary trend	0.0126	0.0139	0.3750
	β_2_	Not detected	−0.1138	0.0877	0.1990	β_2_	Not detected	−0.1879	0.1706	0.2830
	β_3_	Not detected	−0.0027	0.0021	0.2080	β_3_	Not detected	−0.0143	0.0231	0.5410
	Northeast**					Northeast****				
	β_1_	Downward trend	−0.0084	0.0019	0.0001	β_1_	Stationary trend	0.0233	0.0140	0.1170
	β_2_	Abrupt increase	0.2122	0.0543	0.0002	β_2_	Not detected	0.0339	0.1600	0.8348
	β_3_	Progressive decrease	−0.0077	0.0014	0.0000	β_3_	Not detected	−0.0234	0.0114	0.0574
	Southeast***					Southeast***				
	β_1_	Downward trend	−0.0089	0.0019	0.0001	β_1_	Stationary trend	0.0131	0.0097	0.1930
	β_2_	Not detected	0.0776	0.0594	0.1960	β_2_	Not detected	−0.1192	0.1040	0.2650
	β_3_	Progressive decrease	−0.0071	0.0015	0.0000	β_3_	Not detected	−0.0082	0.0143	0.5740
	South**					South*				
	β_1_	Downward trend	−0.0073	0.0023	0.0037	β_1_	Stationary trend	−0.0047	0.0123	0.7040
	β_2_	Not detected	−0.0475	0.0815	0.5610	β_2_	Not detected	0.0158	0.1498	0.9170
	β_3_	Not detected	−0.0008	0.0020	0.6760	β_3_	Not detected	−0.0050	0.0202	0.8070
	Midwest*					Midwest*				
	β_1_	Downward trend	−0.0090	0.0031	0.0060	β_1_	Stationary trend	−0.0243	0.0148	0.1152
	β_2_	Not detected	0.0584	0.0975	0.5509	β_2_	Not detected	−0.0738	0.1857	0.6949
	β_3_	Progressive decrease	−0.0056	0.0023	0.0188	β_3_	Progressive increase	0.0605	0.0237	0.0182
	Brazil***					Brazil****				
	β_1_	Downward trend	−0.0080	0.0012	0.0000	β_1_	Stationary trend	0.0046	0.0050	0.3665
	β_2_	Not detected	0.0622	0.0387	0.1122	β_2_	Not detected	0.0039	0.0703	0.9567
	β_3_	Progressive decrease	−0.0051	0.0009	0.0000	β_3_	Not detected	−0.0063	0.0075	0.4117
Age group (years)	10 to 14***					10 to 14**				
	β_1_	Downward trend	−0.0108	0.0046	0.0233	β_1_	Stationary trend	0.0233	0.0252	0.3660
	β_2_	Not detected	−0.0692	0.1485	0.6426	β_2_	Not detected	−0.1282	0.2737	0.6450
	β_3_	Not detected	−0.0063	0.0036	0.0865	β_3_	Not detected	−0.0259	0.0392	0.5170
	15 to 19 ***					15 to 19**				
	β_1_	Downward trend	−0.0129	0.0028	0.0000	β_1_	Stationary trend	0.0101	0.0152	0.5160
	β_2_	Not detected	0.1686	0.0923	0.0723	β_2_	Not detected	−0.0293	0.2138	0.8920
	β_3_	Progressive decrease	−0.0124	0.0023	0.0000	β_3_	Not detected	0.0078	0.0229	0.7360
	20 to 39***					20 to 29**				
	β_1_	Downward trend	−0.0082	0.0014	0.0000	β_1_	Stationary trend	−0.0018	0.0063	0.7781
	β_2_	Not detected	0.0768	0.0481	0.1152	β_2_	Not detected	0.1169	0.0888	0.2045
	β_3_	Progressive decrease	−0.0043	0.0012	0.0005	β_3_	Not detected	−0.0057	0.0096	0.5637
	40 to 59***					40 to 59*				
	β_1_	Stationary trend	−0.0038	0.0020	0.0673	β_1_	Stationary trend	0.0135	0.0086	0.1282
	β_2_	Not detected	−0.0555	0.0587	0.3481	β_2_	Not detected	−0.1953	0.1039	0.0734
	β_3_	Not detected	−0.0011	0.0015	0.4650	β_3_	Not detected	−0.0055	0.0139	0.6958
	60 or more***					60 or more years**				
	β_1_	Downward trend	−0.0081	0.0029	0.0081	β_1_	Stationary trend	0.0155	0.0154	0.3251
	β_2_	Not detected	0.1511	0.0990	0.1319	β_2_	Not detected	−0.3724	0.2128	0.0972
	β_3_	Progressive decrease	−0.0075	0.0024	0.0033	β_3_	Not detected	0.0387	0.0232	0.1139
Methods	Firearm ***					Firearm***				
	β_1_	Downward trend	−0.0128	0.0017	0.0000	β_1_	Stationary trend	0.0011	0.0053	0.8381
	β_2_	Abrupt increase	0.1308	0.0587	0.0295	β_2_	Not detected	0.0171	0.0747	0.8217
	β_3_	Progressive decrease	−0.0087	0.0014	0.0000	β_3_	Not detected	−0.0034	0.0080	0.6788
	Blunt objects**					Blunt objects*				
	β_1_	Stationary trend	0.0007	0.0014	0.5983	β_1_	Stationary trend	0.0130	0.0072	0.0866
	β_2_	Not detected	−0.0677	0.0522	0.2000	β_2_	Abrupt decrease	−0.2059	0.0876	0.0281
	β_3_	Not detected	0.0013	0.0013	0.3329	β_3_	Not detected	0.0036	0.0117	0.7610
Place of occurrence	At home***					At home***				
	β_1_	Downward trend	−0.004	0.0015	0.0030	β_1_	Stationary trend	0.0015	0.0105	0.8915
	β_2_	Not detected	0.0777	0.0525	0.1437	β_2_	Not detected	0.0494	0.1339	0.7167
	β_3_	Progressive decrease	−0.0037	0.0013	0.0057	β_3_	Not detected	−0.0053	0.0120	0.6619
	Public Space**					Public Space**				
	β_1_	Downward trend	−0.0113	0.0020	0.0000	β_1_	Stationary trend	−0.0021	0.0116	0.8607
	β_2_	Not detected	0.1202	0.0648	0.0682	β_2_	Not detected	0.2069	0.1304	0.1334
	β_3_	Progressive decrease	−0.0081	0.0016	0.0000	β_3_	Not detected	−0.0195	0.0101	0.0708

^a^β (Beta) estimated coefficient from the quasi-Poisson regression model using a log link function, β_1-_pre-intervention trend, β_2-_ represents the immediate level change at pandemic onset, β_3-_ quantifies the slope change following that intervention

^b^SE (Standard Error): standard error of the estimated β, indicating the precision of the coefficient estimate

^c^RR (Rate Ratio): exponential of β (exp(β))

^d^95% CI: 95% confidence interval for the RR, calculated as exp(β ± 1.96 × SE)

*model without seasonality and without lags in the autoregressive process

** model with seasonality and with 1 lag in the autoregressive process (AR(1))

*** model without seasonality and with 1 lag in the autoregressive process (AR(1))

**** model without seasonality and with 2 lag in the autoregressive process (AR(2))

Only the Midwest region retained a significant slope change, while blunt-object homicides displayed a newly emergent abrupt decrease (β₂). For all other variables, point estimates frequently changed direction or moved toward the null, and standard errors widened, indicating reduced precision. This erosion of statistical significance under the shortened observation window suggests that the patterns identified in the main analysis were highly sensitive to the temporal span of pre- and post-intervention segments.

### Placebo analysis

The comparison between the main analysis, using March 2020 as the intervention point, corresponding to the month following confirmation of the first COVID-19 case in Brazil, and the placebo analysis (relocating the intervention to September 2019), revealed heterogeneous patterns of temporal specificity across strata. These findings reinforce that the results should be interpreted as temporal associations rather than causal relationships (Supplementary Material 4).

Under the assumption guiding the placebo test, if the pandemic-attributed impact is genuine, the coefficients for level change (β₂) and/or slope change (β₃) should be statistically significant only in the main analysis, while preserving the direction of the effect, and non-significant in the placebo analysis (Supplementary Material 4).

In several strata, this expected pattern was observed, with statistical significance (*p* < 0.05) in the main analysis, non-significance (*p* > 0.05) in the placebo, and preservation of the effect’s direction. This behavior was identified in the Southeast (β₃, progressive reduction), the Center-West (β₃, progressive reduction), the 15 to 19 year age group (β₃, progressive reduction), and among women aged ≥ 60 years (β₃, progressive reduction). In these cases, the absence of an effect in the placebo analysis, combined with the preservation of the coefficient’s sign, strengthens the plausibility of a temporal association temporally aligned with the onset of the pandemic (Supplementary Material 4).

Conversely, several strata displayed statistically significant coefficients in both the main and placebo analyses, accompanied by reversals in the direction or marked variations in the magnitude of the estimates. This pattern was found in the Northeast (β₂ and β₃), at the national aggregate level (β₃), in the 20–39-year (β₃) and 40–59-year (β₃) age groups, in firearm-related homicides (β₃), and in incidents occurring “at home” and “in public spaces.” In these strata, the negative and significant trend identified in the main analysis (progressive reduction) reversed to a positive and significant trend in the placebo (progressive increase), indicating coefficient instability and lack of temporal specificity (Supplementary Material 4).

Source: Mortality Information System (SIM/SUS) | National Bureau of Statistics (IBGE).

## Discussion

Between January 2017 and December 2022, female homicide rates were highest in Brazil’s most socioeconomically vulnerable regions, particularly the North and Northeast. Adolescents and young adult women were disproportionately affected, firearms predominated as the means of killing, and private residences were the most frequent setting. Adjustments to account for the deterioration in death certificate quality since 2018, driven by the increasing proportion of events of undetermined intent (EUI), were essential to reduce misclassification bias. The magnitude of the increase observed after correction was consistent with that reported by Cerqueira et al. (2025) [15], reinforcing the robustness of the approach. In the Southeast, where EUI deaths were roughly twice as frequent as recorded assaults, the correction increased homicide rates by 28.9%, exceeding changes in other regions and confirming that part of the observed variability in trends could be explained by data quality shifts rather than changes in underlying risk.

Female homicide risk remains disproportionately high in Brazil’s North and Northeast. In 2021, six of the ten states with the highest rates were located in the North, with Roraima recording 7.4 homicides per 100,000 women, 4.93 times the rate observed in São Paulo (1.5 per 100,000) [15]. These regional disparities reflect both demographic and criminological dynamics. The North and Northeast have younger populations, more vulnerable to lethal violence given that violent deaths disproportionately affect younger cohorts. Since the 2000 s, shifts in crime patterns, particularly the spread of violence beyond major urban centers driven by drug trafficking and the expansion of criminal factions, have intensified female homicide rates in these regions [15, 16, 49–54].

Results from the Interrupted Time Series (ITS) analyses indicate that, in most strata, female homicide rates in Brazil were already declining prior to the onset of the COVID-19 pandemic. No statistically significant immediate level change was detected for most variables (*p* > 0.05); however, a significant change in trend was observed in the majority of variables (*p* < 0.05), indicating progressive reductions in female homicide rates during the pandemic.

The absence of a level change in homicide rates at the onset of the pandemic did not signify a reduction in violence against women. Instead, reports of physical and psychological violence increased markedly, accompanied by a rise in hospitalizations for domestic abuse. In Brazil, emergency calls for domestic violence rose by 11.8% during the initial lockdown period (March-May 2020) [26].

At the national level and in most regions, no statistically significant abrupt level shift in female homicides occurred during the pandemic. These findings diverge from patterns documented in Chile, Peru, Mexico, and the city of Rio de Janeiro, where reductions in female homicide, femicide, and attempted femicide were observed over the same period [18–22, 55].

The Northeast region and firearm-related cases were the only strata in Brazil to register an abrupt increase in monthly female homicide rates at the onset of the pandemic. This result warrants cautious interpretation given the methodological constraints of the Interrupted Time Series design, the sensitivity of estimates to the observation period in sensitivity and placebo analyses, and the potential influence of omitted variables.

Despite the aforementioned limitations, it is essential to highlight the persistently high levels of lethal violence in the Northeast. Throughout the study period, this region consistently recorded the highest female homicide rates in Brazil. In 2020 alone, it accounted for 44.0% of all intentional violent deaths in the country and included the three states with the highest overall homicide rates, Ceará, Bahia, and Sergipe [6–8, 15, 16]. This chronic landscape of criminality, underpinned by structural violence and elevated male mortality, fosters a pattern in which femicides are predominantly committed by strangers in public spaces, often linked to summary executions and sexual violence [1, 6, 11, 13, 14, 52].

Firearms play a central role in this dynamic. They constitute the most common means of killing women in Brazil and contribute to both the lethality and geographic spread of gender-based violence, especially in areas dominated by organized crime and armed groups [24]. Within this context, it is worth emphasizing that, between 2019 and 2022, measures were implemented that weakened the mechanisms for controlling and monitoring firearms and ammunition [14, 15, 52]. Such deregulation likely exacerbated the risk of domestic violence, femicide, suicide, and firearm-related accidents involving children, as documented by several studies [55, 56]. It is estimated that, had the federal government not relaxed the provisions of the Disarmament Statute, at least 6,379 lives could have been saved between 2019 and 2021 [15].

Throughout the COVID-19 pandemic in Brazil, a trend shift was observed, characterized by a progressive decline in monthly female homicide rates across most regions, age groups, settings (domestic and public), and firearm-related homicides. This pattern contrasts with findings from other Latin American countries, where initial reductions were followed by a resurgence in rates, ultimately returning to pre-pandemic levels [18–22].

One plausible explanation for Brazil’s downward trend is a reduction in lethal violence within domestic settings, potentially supplanted by an increase in non-lethal forms of aggression [18–22, 26]. Concurrently, lockdown measures, including restrictions on mobility and the closure of bars, commercial establishments, and recreational venues, may have sharply curtailed women’s exposure to violence in public spaces, whether perpetrated by intimate partners or strangers [19,21,57,62].

Importantly, the decline observed during the pandemic appears to reinforce a pre-existing downward trajectory that began after the 2016–2017 peak in national homicide rates, a period marked by intense conflicts between criminal factions and territorial disputes [15, 16, 51]. From 2018 onward, this decline persisted, albeit with regional variation. This period also coincided with documented deterioration in death certificate quality; despite correction procedures, residual misclassification cannot be fully ruled out [15, 16, 51].

Despite the observed reduction in female homicide rates over the analyzed period, Brazil remains a violent country for women, as national rates continue to exceed 3.0 per 100,000 women. In the North and Northeast regions, homicide levels are comparable to those recorded in Guatemala and El Salvador, two of the most violent countries for women in Latin America and the Caribbean [47, 48].

Building on this regional perspective, national data indicate that between 2013 and 2023 Brazil registered 47,463 female homicides, corresponding to an average of 13 deaths per day. In 2023 alone, 3,903 cases were reported, resulting in a rate of 3.5 per 100,000 women. Although the country experienced an overall decline in homicide rates during this period, the reduction was less pronounced among women. Furthermore, the stagnation observed between 2022 and 2023 reveals that lethal violence against women has not mirrored the downward trend documented in the general population [32].

This persistence becomes even more concerning when considering the specific phenomenon of femicide. Estimates suggest that approximately one-third of female homicides fall into this category. In 2024, 1,492 femicides were officially recorded, equivalent to 35.2% of all female homicides [16].

The magnitude of these figures demonstrates that the problem is not limited to isolated cases of violence but reflects a systemic and gendered pattern of lethal victimization. The persistence of high female homicide rates, combined with the substantial proportion of femicides, underscores the urgent need for integrated public policies. Such strategies must coordinate public security, the justice system, and social protection networks. Addressing this phenomenon requires not only reinforcing existing legal and institutional mechanisms but also dismantling the structural inequalities that sustain gender-based lethal violence in Brazil [15, 16, 32–35].

### Limitations and implications of the study

The primary limitation of this study concerns the quality of mortality data in Brazil. Over the study period, deaths classified as events of undetermined intent (EUI) increased, potentially biasing estimates of female homicides. To mitigate misclassification bias, we applied corrections to death records using methodologies previously adopted in Brazilian studies [15, 38]. This approach assumes that EUI are proportionally distributed across the demographic and temporal profiles of other external causes within each stratum (year, month, age group). If particular subgroups are more likely to be misclassified as EUI, differential misclassification may occur. Even so, proportional redistribution yields more accurate homicide estimates than leaving the data uncorrected [36].

Empirical evidence supports this correction. Souza et al. (2017) documented inverse trends between female homicides and EUI from 1980 to 2014, indicating that declines in recorded homicides coincided with increases in EUI classifications [34]. Similarly, Borges et al. (2019) showed that EUI inflates or suppresses period effects, with higher homicide risk mirroring lower EUI risk and vice versa [35]. Together, these findings justify the use of proportional redistribution in time-series analyses of homicide trends.

This study is also subject to limitations intrinsic to the interrupted time-series (ITS) design, including the absence of monthly covariates, incomplete verification of pre-intervention trend stability, and the possibility that concurrent, unmeasured shocks influenced outcomes. Sensitivity analyses showed that estimated level and slope changes were highly dependent on the temporal window, with loss of statistical significance and coefficient instability when pre- and post-intervention periods were shortened. Placebo analyses revealed inconsistent temporal specificity across strata, with some preserving the expected pattern and others exhibiting significant effects in the opposite direction across models. These results point to potential residual confounding and reinforce interpreting the observed changes as temporal associations, not causal effects [32, 38–40].

Despite these limitations, examining the temporal association between the COVID-19 pandemic and female homicide rates offers important insights into how a large-scale health crisis may intersect with patterns of lethal violence against women. The findings contribute to the evidence base needed to inform public policies and prevention strategies sensitive to regional, demographic, and situational differences.

## Conclusions

This study demonstrates that, between January 2017 and December 2022, Brazil exhibited regional disparities in monthly female homicide rates, with the North and Northeast bearing the greatest burden of lethal gender-based violence. Adolescents and young adult women were disproportionately affected, firearms predominated as the means of killing, and private residences were the most frequent setting.

The Northeast region and firearm-related cases were the only categories to show an abrupt increase in female homicide rates at the onset of the pandemic. Moreover, our Interrupted Time Series (ITS) analyses revealed that, although female homicides decreased during the COVID-19. The Northeast region and firearm-related cases were the only categories to show an abrupt increase in female homicide rates at the onset of the pandemic. Moreover, our Interrupted Time Series (ITS) analyses revealed that, although female homicides decreased during the COVID-19 pandemic, these findings warrant cautious interpretation. Sensitivity and placebo analyses indicated that the results were influenced by the choice of observation window and may be partially explained by omitted variables, such as shifts in crime dynamics, law enforcement activity, or reporting practices. The evidence supports interpreting these results as temporal associations rather than causal effects of the pandemic itself.

Nonetheless, persistently high female homicide rates in Brazil, particularly in the Northeast, highlight the need to strengthen mortality surveillance, improve misclassification corrections, and adopt region-specific prevention strategies, including firearm control, protective services, and targeted social policies. These elevated rates are concentrated in regions with greater socioeconomic vulnerability, the expansion of organized crime and drug trafficking, and limited availability of protection and assistance services for women experiencing violence, especially in the Northeast and North. In this context, addressing regional disparities is not only crucial for reducing localized risks but also essential for shaping comprehensive national strategies that integrate public security, social protection, health service, and gender-based violence prevention.

## Supplementary Information

Supplementary Material 1.

Supplementary Material 2.

Supplementary Material 3.

Supplementary Material 4.

## Data Availability

The data are provided within the manuscript or supplementary information files.

## Abbreviations

ACF: Autocorrelation Function
AR: Autoregressive
COVID-19: Coronavirus Disease 2019
DATASUS: Department of Informatics of the Brazilian Unified Health System
EUI: Events of Undetermined Extent
GATHER: Guidelines for Accurate and Transparent Health Estimates Reporting
IBGE: Instituto Brasileiro de Geografia e Estatística
RAIQ: Rates adjusted for Information Quality
RAIQC: Rates Adjusted for Information Quality and Coverage
ICD-10: International Classification of Diseases
ITS: Interrupted time series
95% CI: 95% Confidence Intervals
LAC: Latin America and the Caribbean
LOESS: Locally Estimated Scatterplot Smoothing
PACF: Partial Autocorrelation Function Plots
RR: Relative Risk
SIM: Mortality Information System
SARS-CoV-2: Severe Acute Respiratory Syndrome Coronavirus 2
UR: Uncorrected Rates
WHO: World Health Organization
