# Supplementary material for: Female homicides in Brazil before and during the COVID-19 pandemic: an interrupted time-series analysis
Source: BMC Public Health. 2025 Oct 24;25:3597. doi: 10.1186/s12889-025-24814-6 (PMC12553206; doi:10.1186/s12889-025-24814-6)
Supplement: Supplementary file 1 — Supplementary Material 1. [file 12889_2025_24814_MOESM1_ESM.docx]

Supplementary Material 1- Number of death records due to accidental external causes, assault, self-inflicted, event whose intention is undetermined and legal intervention in women, from January 2017 to December 2022, Brazil, 2025.

| **Methods** | | | | | | | | |
| --- | --- | --- | --- | --- | --- | --- | --- | --- |
| **Firearm** | | | | | | | | |
| **Year** | **W32 to W34** | **X72 to X74** | **X93 to X95** | **Y22 to Y24** | **Y35** | **%EUI no TEC** | **% homicides in TEC*** | **Firearm homicide corrected** |
| 2017 | 20 | 111 | 2,593 | 39 | 8 | 1.43 | 94.91 | 2,630 |
| 2018 | 14 | 97 | 2,286 | 59 | 6 | 2.46 | 95.13 | 2,342 |
| 2019 | 21 | 89 | 1,783 | 91 | 9 | 4.78 | 93.74 | 1,868 |
| 2020 | 19 | 105 | 1,881 | 65 | 11 | 3.22 | 93.30 | 1,942 |
| 2021 | 15 | 97 | 1,880 | 60 | 14 | 2.99 | 93.72 | 1,936 |
| 2022 | 15 | 100 | 1,851 | 60 | 18 | 3.02 | 93.30 | 1,907 |
| Total | 104 | 599 | 12,274 | 374 | 66 | 2.87 | 94.10 | 12,626 |
| **Blunt objects** | | | | | | | | |
| **Year** | **W25 to W26** | **X78 to X79** | **X99 to Y00** | **Y28 to Y29** | **Y35** | **%EUI no TEC** | **% homicide in TEC*** | **Blunt object homicides corrected** |
| 2017 | 1 | 51 | 1,463 | 201 | 8 | 13.20 | 96.06 | 1,656 |
| 2018 | 8 | 32 | 1,392 | 403 | 6 | 28.03 | 96.80 | 1,782 |
| 2019 | 7 | 53 | 1,246 | 426 | 9 | 32.40 | 94.75 | 1,650 |
| 2020 | 4 | 56 | 1,211 | 450 | 11 | 35.10 | 94.46 | 1,636 |
| 2021 | 4 | 59 | 1,226 | 501 | 14 | 38.45 | 94.09 | 1,697 |
| 2022 | 3 | 47 | 1,239 | 545 | 18 | 41.70 | 94.80 | 1,756 |
| Total | 27 | 298 | 7,777 | 2,526 | 66 | 30.93 | 95.21 | 10,182 |
| **Place of occurrence** | | | | | | | | |
| **At home** | | | | | | | | |
| **Year** | **W00 to X59** | **X60 to X84** | **X85 to Y09** | **Y10 to Y34** | **Y35** | **%EUI no TEC** | **% homicide in TEC*** | **Homicides corrected** |
| 2017 | 1151 | 1586 | 1349 | 422 | 1 | 10.33 | 33.02 | 1,488 |
| 2018 | 1190 | 1642 | 1267 | 501 | 0 | 12.22 | 30.91 | 1,422 |
| 2019 | 1178 | 1780 | 1154 | 598 | 2 | 14.54 | 28.05 | 1,322 |
| 2020 | 1420 | 1765 | 1173 | 665 | 1 | 15.26 | 26.91 | 1,352 |
| 2021 | 1523 | 2057 | 1189 | 700 | 1 | 14.68 | 24.91 | 1,363 |
| 2022 | 1658 | 2078 | 1174 | 755 | 3 | 15.37 | 23.91 | 1,354 |
| Total | 8120 | 10908 | 7306 | 3641 | 8 | 13.82 | 27.74 | 8,316 |
| **Public space** | | | | | | | | |
| **Year** | **W00 to X59** | **X60 to X84** | **X85 to Y09** | **Y10 to Y34** | **Y35** | **%EUI no TEC** | **% homicide in TEC*** | **Homicides corrected** |
| 2017 | 90 | 90 | 1405 | 119 | 3 | 7.49 | 88.48 | 1,503 |
| 2018 | 85 | 97 | 1338 | 150 | 0 | 9.87 | 88.03 | 1,459 |
| 2019 | 84 | 108 | 973 | 165 | 1 | 14.15 | 83.45 | 1,100 |
| 2020 | 102 | 110 | 1034 | 165 | 2 | 13.22 | 82.85 | 1,163 |
| 2021 | 75 | 115 | 1012 | 204 | 2 | 16.94 | 84.05 | 1,167 |
| 2022 | 126 | 116 | 977 | 199 | 5 | 16.26 | 79.82 | 1,127 |
| Total | 562 | 636 | 6739 | 1002 | 13 | 12.60 | 84.77 | 7,588 |
| **Locality** | | | | | | | | |
| **Brazil** | | | | | | | | |
| **Year** | **W00 to X59** | **X60 to X84** | **X85 to Y09** | **Y10 to Y34** | **Y35** | **%EUI no TEC** | **% homicide in TEC*** | **Homicides corrected** |
| 2017 | 10197 | 2659 | 4754 | 2418 | 8 | 13.72 | 26.98 | 5406 |
| 2018 | 10301 | 2726 | 4320 | 2961 | 6 | 17.06 | 24.89 | 5057 |
| 2019 | 10289 | 2919 | 3581 | 3609 | 9 | 21.48 | 21.32 | 4350 |
| 2020 | 9953 | 2963 | 3695 | 3505 | 11 | 21.09 | 22.23 | 4474 |
| 2021 | 11227 | 3424 | 3717 | 3811 | 14 | 20.73 | 20.22 | 4488 |
| 2022 | 12522 | 3552 | 3660 | 4022 | 18 | 20.36 | 18.53 | 4405 |
| Total | 64489 | 18243 | 23727 | 20326 | 66 | 19.08 | 22.27 | 28254 |
| **North** | | | | | | | | |
| **Year** | **W00 to X59** | **X60 to X84** | **X85 to Y09** | **Y10 to Y34** | **Y35** | **%EUI no TEC** | **% homicide in TEC*** | **Homicides corrected** |
| 2017 | 474 | 202 | 581 | 33 | 1 | 2.62 | 46.18 | 596 |
| 2018 | 431 | 226 | 598 | 61 | 0 | 4.86 | 47.65 | 627 |
| 2019 | 450 | 231 | 469 | 110 | 2 | 9.55 | 40.71 | 514 |
| 2020 | 436 | 237 | 426 | 102 | 1 | 9.27 | 38.73 | 466 |
| 2021 | 491 | 267 | 486 | 92 | 1 | 7.39 | 39.04 | 522 |
| 2022 | 518 | 304 | 453 | 85 | 0 | 6.67 | 35.53 | 483 |
| Total | 2800 | 1467 | 3013 | 483 | 8 | 6.63 | 41.34 | 3213 |
| **Northeast** | | | | | | | | |
| **Year** | **W00 to X59** | **X60 to X84** | **X85 to Y09** | **Y10 to Y34** | **Y35** | **%EUI no TEC** | **% homicide in TEC*** | **Homicides corrected** |
| 2017 | 2366 | 589 | 1730 | 660 | 2 | 14.08 | 36.91 | 1899 |
| 2018 | 2295 | 594 | 1567 | 804 | 0 | 18.04 | 35.17 | 1779 |
| 2019 | 2335 | 616 | 1258 | 955 | 0 | 22.69 | 29.89 | 1469 |
| 2020 | 2334 | 639 | 1448 | 959 | 4 | 21.67 | 32.72 | 1686 |
| 2021 | 2481 | 757 | 1450 | 989 | 6 | 21.07 | 30.89 | 1652 |
| 2022 | 2772 | 795 | 1347 | 1059 | 0 | 21.55 | 27.41 | 1552 |
| Total | 14583 | 3990 | 8800 | 5426 | 14 | 19.81 | 32.13 | 10045 |
| **Southeast** | | | | | | | | |
| **Year** | **W00 to X59** | **X60 to X84** | **X85 to Y09** | **Y10 to Y34** | **Y35** | **%EUI no TEC** | **% homicide in TEC*** | **Homicides corrected** |
| 2017 | 4760 | 999 | 1367 | 1556 | 3 | 21.83 | 19.18 | 1665 |
| 2018 | 4797 | 1024 | 1204 | 1876 | 6 | 26.68 | 17.12 | 1525 |
| 2019 | 4687 | 1117 | 968 | 2294 | 5 | 33.85 | 14.28 | 1296 |
| 2020 | 4550 | 1171 | 1001 | 2084 | 6 | 30.98 | 14.88 | 1311 |
| 2021 | 5319 | 1338 | 949 | 2296 | 4 | 30.17 | 12.47 | 1235 |
| 2022 | 5854 | 1342 | 955 | 2444 | 7 | 29.96 | 11.71 | 1241 |
| Total | 29967 | 6991 | 6444 | 12550 | 31 | 28.90 | 14.84 | 8306 |
| **South** | | | | | | | | |
| **Year** | **W00 to X59** | **X60 to X84** | **X85 to Y09** | **Y10 to Y34** | **Y35** | **%EUI no TEC** | **% homicide in TEC*** | **Homicides corrected** |
| 2017 | 1759 | 612 | 640 | 101 | 1 | 3.35 | 21.25 | 661 |
| 2018 | 1950 | 598 | 543 | 134 | 0 | 4.34 | 17.57 | 567 |
| 2019 | 2007 | 653 | 538 | 146 | 0 | 4.57 | 16.82 | 563 |
| 2020 | 1899 | 637 | 483 | 214 | 0 | 7.09 | 16.00 | 517 |
| 2021 | 2107 | 726 | 502 | 239 | 3 | 7.16 | 15.04 | 538 |
| 2022 | 2375 | 733 | 563 | 268 | 5 | 7.29 | 15.32 | 604 |
| Total | 12097 | 3959 | 3269 | 1102 | 9 | 5.70 | 16.91 | 3455 |
| **Midwest** | | | | | | | | |
| **Year** | **W00 to X59** | **X60 to X84** | **X85 to Y09** | **Y10 to Y34** | **Y35** | **%EUI no TEC** | **% homicide in TEC*** | **Homicides corrected** |
| 2017 | 838 | 257 | 436 | 68 | 1 | 4.44 | 28.46 | 455 |
| 2018 | 828 | 284 | 408 | 86 | 0 | 5.66 | 26.84 | 431 |
| 2019 | 810 | 302 | 348 | 104 | 0 | 7.12 | 23.84 | 373 |
| 2020 | 734 | 279 | 337 | 146 | 0 | 10.81 | 24.96 | 373 |
| 2021 | 829 | 336 | 330 | 195 | 0 | 13.04 | 22.07 | 373 |
| 2022 | 1003 | 378 | 342 | 166 | 3 | 9.62 | 19.81 | 375 |
| Total | 5042 | 1836 | 2201 | 765 | 4 | 8.42 | 24.23 | 2386 |

Note: ^a^TEC: Total accidental external causes, self-inflicted, assaults and legal interventions; ^b^**∆%:** percentage change in total corrected homicides compared to homicides officially registered in the Mortality Information System. Code in the International Statistical Classification of Diseases and Related Health Problems:X85–Y09: Homicides (assaults);X93–X95: Firearm-related homicides;X99–Y00: Blunt objects; andY35: Legal intervention Y10–Y34: Undetermined intent;Y22–Y24: Firearm-related injuries of undetermined intent;Y28–Y29: Blunt objects of undetermined intent; X60 to X84 :Self-inflicted injuries; W00 to X59: Accidental trauma; X78 to X79: Self-inflicted injuries by Blunt object; W25 to W26: Accidental trauma by sharp, penetrating, or piercing object; X72 to X74: Self-inflicted firearm injuries; W32 to W34: Accidental firearm trauma.
