# Supplementary material for: Female homicides in Brazil before and during the COVID-19 pandemic: an interrupted time-series analysis
Source: BMC Public Health. 2025 Oct 24;25:3597. doi: 10.1186/s12889-025-24814-6 (PMC12553206; doi:10.1186/s12889-025-24814-6)
Supplement: Supplementary file 2 — Supplementary Material 2. [file 12889_2025_24814_MOESM2_ESM.docx]

**Supplementary Material 2. Steps for correcting female homicide records according to the method proposed by Garcia et al. (2016)[39].**

| **Total Homicides of Women in Public Spaces** |
| --- |
| **ICD-10 Codes** |
| Homicides of women in public spaces: X85 to Y09 |
| Self-inflicted injuries of women in public spaces: X60 to X84 |
| Accidental trauma in women in public spaces: W00 to X59 |
| Legal intervention in public spaces: Y35^a^ |
| Events of undetermined intent involving women in public spaces: Y10 to Y34 |
| **Data Quality Adjustment** |
| **Proportional Redistribution by Month and Age Group** |
| **Phase 1:** Divide the total number of deaths classified as X85 to Y09 by (X85 to Y09 + W00 to X59 + X60 to X84 + Y35). |
| **Phase 2:** Multiply the result from phase 1 by the mortality records classified as Y10 to Y34. |
| **Phase 3:** Sum the result from phase 2 with the mortality records classified as X85 to Y09. |
| **Total Homicides of Women in Residential Settings** |
| **ICD-10 Codes** |
| Homicides of women in residential settings: X85 to Y09 |
| Self-inflicted injuries of women in residential settings: X60 to X84 |
| Accidental trauma in women in residential settings: W00 to X59 |
| Legal intervention involving women in residential settings: Y35 |
| Events of undetermined intent involving women in residential settings: Y10 to Y34 |
| **Data Quality Adjustment** |
| **Proportional Redistribution by Month and Age Group** |
| **Phase 1:** Divide the total number of deaths classified as X85 to Y09 by (X85 to Y09 + W00 to X59 + X60 to X84 + Y35). |
| **Phase 2:** Multiply the result from phase 1 by the mortality records classified as Y10 to Y34. |
| **Phase 3:** Sum the result from phase 2 with the mortality records classified as X85 to Y09. |
| **Homicides of Women by Firearm** |
| **ICD-10 Codes** |
| Female homicides by firearm: X93 to X95 |
| Self-inflicted firearm injuries in women: X72 to X74 |
| Accidental firearm trauma in women: W32 to W34 |
| Legal intervention involving women: Y35 |
| Firearm-related events of undetermined intent in women: Y22, Y23, Y24 |
| **Data Quality Adjustment** |
| **Proportional Redistribution by Month and Age Group** |
| **Phase 1:** Divide the total number of deaths classified as X93 to X95 by (X93 to X95 + W32 to W34 + X72 to X74 + Y35). |
| **Phase 2:** Multiply the result from phase 1 by the mortality records classified as Y22, Y23, Y24 |
| **Phase 3:** Sum the result from phase 2 with the mortality records classified as X93 to X95. |
| **Homicides of Women by Blunt Object** |
| **ICD-10 Codes** |
| Homicide of women by Blunt object: X99 to Y00 |
| Self-inflicted injuries in women by sBlunt object: X78 to X79 |
| Accidental trauma in women by sharp, penetrating, or piercing object: W25 to W26 |
| Legal intervention involving women: Y35 |
| Events involving Blunt object of undetermined intent in women: Y28, Y29 |
| **Data Quality Adjustment** |
| **Proportional Redistribution by Month and Age Group** |
| **Phase 1:** Divide the total number of deaths classified as X99 to Y00 by (X99 to Y00 + W25 to W26 + X78 to X79 + Y35). |
| **Phase 2:** Multiply the result from phase 1 by the mortality records classified as Y28, Y29 |
| **Phase 3:** Sum the result from phase 2 with the mortality records classified as X99 and Y009. |

Note:^a^Y35-The ICD-10 code Y35, designated as *Legal intervention*, refers to deaths resulting from the use of force by state agents acting in the course of duty, such as during police operations, arrests, or crowd control. Scholars argue that Y35 may function as a mechanism of institutional opacity, potentially concealing extrajudicial killings and shielding state actors from accountability. The use of this category can obscure patterns of lethal state violence, particularly in racially and socioeconomically marginalized populations. As such, Y35 constitutes a crucial analytical tool for examining police lethality and the broader dynamics of state-sanctioned violence.
