# Supplementary material for: Female homicides in Brazil before and during the COVID-19 pandemic: an interrupted time-series analysis
Source: BMC Public Health. 2025 Oct 24;25:3597. doi: 10.1186/s12889-025-24814-6 (PMC12553206; doi:10.1186/s12889-025-24814-6)
Supplement: Supplementary file 3 — Supplementary Material 3. [file 12889_2025_24814_MOESM3_ESM.docx]

Supplementary Material 3-Linearity assessment of pre-pandemic female-homicide trends (January 2017 to February 2020) using quasi-Poisson regression: comparison of linear, quadratic and cubic models via quasi-F test and ΔQAIC.

| **Locality** | **dev_lin** | **df_lin** | **dev_quad** | **df_quad** | **dev_cub** | **df_cub** | **p-valor (lin vs quad)** | **p- valor (quad vs cub)** | **p-valor (lin vs.cub)** | **QAIC_lin** | **QAIC_quad** | **QAIC_cub** |
| --- | --- | --- | --- | --- | --- | --- | --- | --- | --- | --- | --- | --- |
| North | 60.7 | 36.0 | 57.2 | 35.0 | 51.4 | 34.0 | 0.151 | 0.0611 | 0.0617 | 67.5 | 67.0 | 63.7 |
| Northeast | 82.7 | 36.0 | 82.1 | 35.0 | 50.1 | 34.0 | 0.617 | <0.001 | <0.001 | 92.0 | 96.3 | 61.9 |
| Southeast | 75.7 | 36.0 | 74.9 | 35.0 | 69.1 | 34.0 | 0.566 | 0.0972 | 0.21 | 84.0 | 87.7 | 85.2 |
| South | 44.9 | 36.0 | 43.3 | 35.0 | 43.0 | 34.0 | 0.263 | 0.613 | 0.473 | 49.9 | 50.7 | 53.0 |
| Midwest | 53.0 | 36.0 | 49.7 | 35.0 | 49.7 | 34.0 | 0.141 | 0.987 | 0.344 | 59.0 | 58.3 | 61.5 |
| Brazil | 101 | 36 | 100 | 35 | 71.7 | 34 | 0.677 | <0.001 | 0.00305 | 112 | 118 | 88.7 |
| **Age groups (years)** | **dev_lin** | **df_lin** | **dev_quad** | **df_quad** | **dev_cub** | **df_cub** | **p-valor (lin vs quad)** | **p- valor (quad vs cub)** | **p-valor (lin vs.cub)** | **QAIC_lin** | **QAIC_quad** | **QAIC_cub** |
| 10 to 14 | 37.9 | 36.0 | 37.7 | 35.0 | 37.4 | 34.0 | 0.7080 | 0.5780 | 0.7980 | 43.8 | 44.6 | 45.4 |
| 15 to 19 | 59.8 | 36.0 | 57.3 | 35.0 | 43.8 | 34.0 | 0.2350 | 0.0024 | 0.0046 | 39.2 | 40.2 | 42.6 |
| 20 to 39 | 67.1 | 36.0 | 65.7 | 35.0 | 55.5 | 34.0 | 0.3890 | 0.0174 | 0.0392 | 39.9 | 40.9 | 42.0 |
| 40 to 59 | 67.2 | 36.0 | 66.7 | 35.0 | 63.7 | 34.0 | 0.6320 | 0.2040 | 0.3950 | 40.8 | 41.7 | 42.6 |
| 60 or more | 43.5 | 36.0 | 43.3 | 35.0 | 42.7 | 34.0 | 0.6950 | 0.4780 | 0.7180 | 39.9 | 41.0 | 42.0 |
| **Methods** | **dev_lin** | **df_lin** | **dev_quad** | **df_quad** | **dev_cub** | **df_cub** | **p-valor (lin vs quad)** | **p- valor (quad vs cub)** | **p-valor (lin vs.cub)** | **QAIC_lin** | **QAIC_quad** | **QAIC_cub** |
| Firearm | 87.2 | 36 | 86.1 | 35 | 56.6 | 34 | 0.5090 | 0.0010 | 0.0010 | 97.2 | 101 | 70.2 |
| Blunt objects | 43.9 | 36 | 43.3 | 35 | 42.1 | 34 | 0.4860 | 0.3380 | 0.4940 | 48.9 | 50.8 | 52.1 |
| **Place of occurrence** | **dev_lin** | **df_lin** | **dev_quad** | **df_quad** | **dev_cub** | **df_cub** | **p-valor (lin vs quad)** | **p- valor (quad vs cub)** | **p-valor (lin vs.cub)** | **QAIC_lin** | **QAIC_quad** | **QAIC_cub** |
| Public Space | 71.3 | 36 | 68.4 | 35 | 58.9 | 34 | 0.2330 | 0.0257 | 0.0400 | 79.2 | 80.2 | 72.9 |
| At home | 43.9 | 36 | 43.6 | 35 | 39.6 | 34 | 0.5830 | 0.0721 | 0.1670 | 48.9 | 51.1 | 48.9 |

Note: dev_lin- residual quasi-deviance of the linear model; df_lin- degrees of freedom of the linear model; dev_quad- residual quasi-deviance of the quadratic model; df_quad- degrees of freedom of the quadratic model; dev_cub- residual quasi-deviance of the cubic model; df_cub- degrees of freedom of the cubic model; p-valor lin vs quad- p-values arise from quasi-F tests that compare, respectively, the linear versus quadratic (H_0_: linear model fitted the data better than the quadratic model); p- valor quad vs cub- p-values arise from quasi-F tests that compare, respectively, the quadradic versus cubic (H_0_: quadradic model fitted the data better than the cubic model); p-valor lin vs.cub**-** p-values arise from quasi-F tests that compare, respectively, the linear versus cubic (H_0_: linear model fitted the data better than the cubic model); QAIC_lin- Quasi-Akaike Information Criterion (QAIC) for the linear model; QAIC_quad -Quasi-Akaike Information Criterion (QAIC) for the quadratic model; QAIC_cub- Quasi-Akaike Information Criterion (QAIC) for the cubic model

-
