# Supplementary material for: Female homicides in Brazil before and during the COVID-19 pandemic: an interrupted time-series analysis
Source: BMC Public Health. 2025 Oct 24;25:3597. doi: 10.1186/s12889-025-24814-6 (PMC12553206; doi:10.1186/s12889-025-24814-6)
Supplement: Supplementary file 4 — Supplementary Material 4. [file 12889_2025_24814_MOESM4_ESM.docx]

Supplementary Material 4- Placebo analysis of the interrupted time series model, comparing the beginning of the pandemic in March 2020, with the beginning in September 2019

| **BP (Jan 2017 to Feb 2020) and DP ( Marc 2020 to Dec 2022)** | | | | | | **Placebo analysis- BP (Jan 2017 to Aug 2019) DP (Sep 2019 to Dec 2022)** | | | | |
| --- | --- | --- | --- | --- | --- | --- | --- | --- | --- | --- |
| **Variable** | **Categories** | **Interpretation** | **β** | **SE** | **p-value** | **Categories** | **Interpretation** | **β** | **SE** | **p-value** |
| Locality | North* |  |  |  |  | North* |  |  |  |  |
|  | β2 | Not detected | -0.1138 | 0.0877 | 0.1990 | β2 | Not detected | -0.0640 | 0.0953 | 0.5040 |
|  | β3 | Not detected | -0.0027 | 0.0021 | 0.2080 | β3 | Not detected | 0.0030 | 0.0046 | 0.5130 |
|  | Northeast** |  |  |  |  | Northeast** |  |  |  |  |
|  | β2 | Abrupt increase | 0.2122 | 0.0543 | **0.0002** | β2 | Abrupt increase | 0.1836 | 0.0543 | **0.0012** |
|  | β3 | Progressive decrease | -0.0077 | 0.0014 | **0.0000** | β3 | Progressive increase | 0.0105 | 0.0029 | **0.0005** |
|  | Southeast*** |  |  |  |  | Southeast*** |  |  |  |  |
|  | β2 | Not detected | 0.0776 | 0.0594 | 0.1960 | β2 | Not detected | 0.0784 | 0.0605 | 0.1993 |
|  | β3 | Progressive decrease | -0.0071 | 0.0015 | **0.0000** | β3 | Not detected | 0.0057 | 0.0031 | 0.0710 |
|  | South** |  |  |  |  | South** |  |  |  |  |
|  | β2 | Not detected | -0.0475 | 0.0815 | 0.5610 | β2 | Not detected | -0.0377 | 0.0785 | 0.6322 |
|  | β3 | Not detected | -0.0008 | 0.0020 | 0.6760 | β3 | Progressive increase | 0.0116 | 0.0037 | **0.0029** |
|  | Midwest* |  |  |  |  | Midwest* |  |  |  |  |
|  | β2 | Not detected | 0.0584 | 0.0975 | 0.5509 | β2 | Not detected | -0.1554 | 0.1007 | 0.1275 |
|  | β3 | Progressive decrease | -0.0056 | 0.0023 | **0.0188** | β3 | Not detected | 0.0088 | 0.0049 | 0.0773 |
|  | Brazil*** |  |  |  |  | Brazil** |  |  |  |  |
|  | β1 | Downward trend | -0.0080 | 0.0012 | **0.0000** | β1 | Downward trend | -0.0101 | 0.0019 | **0.0000** |
|  | β2 | Not detected | 0.0622 | 0.0387 | 0.1122 | β2 | Not detected | 0.0717 | 0.0386 | 0.0677 |
|  | β3 | Progressive decrease | -0.0051 | 0.0009 | **0.0000** | β3 | Progressive increase | 0.0084 | 0.0022 | **0.0002** |
| Age group (years) | 10 to 14*** |  |  |  |  | 10 to 14*** |  |  |  |  |
|  | β2 | Not detected | -0.0692 | 0.1485 | 0.6426 | B2 | Not detected | 0.1251 | 0.1471 | 0.3979 |
|  | β3 | Not detected | -0.0063 | 0.0036 | 0.0865 | B3 | Not detected | 0.0077 | 0.0074 | 0.3000 |
|  | 15 to 19 *** |  |  |  |  | 15 to 19*** |  |  |  |  |
|  | β1 | Downward trend | -0.0129 | 0.0028 | **0.0000** | β1 | Downward trend | -0.0092 | 0.0041 | **0.0291** |
|  | β2 | Not detected | 0.1686 | 0.0923 | 0.0723 | β2 | Not detected | 0.0309 | 0.0933 | 0.7412 |
|  | β3 | Progressive decrease | -0.0124 | 0.0023 | **0.0000** | β3 | Not detected | 0.0037 | 0.0047 | 0.4333 |
|  | 20 to 39*** |  |  |  |  | 20 to 29*** |  |  |  |  |
|  | β2 | Not detected | 0.0768 | 0.0481 | 0.1152 | β2 | Not detected | -0.0005 | 0.0420 | 0.9900 |
|  | β3 | Progressive decrease | -0.0043 | 0.0012 | **0.0005** | β3 | Progressive increase | 0.0109 | 0.0024 | **0.0000** |
|  | 40 to 59*** |  |  |  |  | 40 to 59**** |  |  |  |  |
|  | β1 | Stationary trend | -0.0038 | 0.0020 | 0.0673 | β1 | Downward trend | -0.0076 | 0.0025 | **0.0030** |
|  | β2 | Not detected | -0.0555 | 0.0587 | 0.3481 | β2 | Not detected | -0.0117 | 0.0555 | 0.8334 |
|  | β3 | Not detected | -0.0011 | 0.0015 | 0.4650 | β3 | Progressive increase | 0.0087 | 0.0030 | **0.0049** |
|  | 60 or more*** |  |  |  |  | 60 or more**** |  |  |  |  |
|  | β2 | Not detected | 0.1511 | 0.0990 | 0.1319 | β2 | Not detected | 0.0675 | 0.1038 | 0.5179 |
|  | β3 | Progressive decrease | -0.0075 | 0.0024 | **0.0033** | β3 | Not detected | 0.0053 | 0.0054 | 0.3320 |
| Methods | Firearm *** |  |  |  |  | Firearm*** |  |  |  |  |
|  | β2 | Abrupt increase | 0.1308 | 0.0587 | 0.0295 | β2 | Not detected | -0.0117 | 0.0515 | 0.8209 |
|  | β3 | Progressive decrease | -0.0087 | 0.0014 | **0.0000** | β3 | Progressive increase | 0.0121 | 0.0030 | **0.0001** |
|  | Blunt objects** |  |  |  |  | Blunt objects** |  |  |  |  |
|  | β2 | Not detected | -0.0677 | 0.0522 | 0.2000 | β2 | Not detected | -0.0593 | 0.0504 | 0.2444 |
|  | β3 | Not detected | 0.0013 | 0.0013 | 0.3329 | β3 | Not detected | 0.0004 | 0.0025 | 0.8710 |
| Place of occurrence | At home*** |  |  |  |  | At home**** |  |  |  |  |
|  | β2 | Not detected | 0.0777 | 0.0525 | 0.1437 | β2 | Not detected | 0.0339 | 0.0521 | 0.5180 |
|  | β3 | Progressive decrease | -0.0037 | 0.0013 | **0.0057** | β3 | Progressive increase | 0.0061 | 0.0028 | **0.0296** |
|  | Public Space** |  |  |  |  | Public Space*** |  |  |  |  |
|  | β2 | Not detected | 0.1202 | 0.0648 | 0.0682 | β2 | Not detected | 0.0080 | 0.0615 | 0.8879 |
|  | β3 | Progressive decrease | -0.0081 | 0.0016 | **0.0000** | β3 | Progressive increase | 0.0091 | 0.0034 | **0.0093** |

Note: ^a^β (Beta): estimated coefficient from the quasi-Poisson regression model using a log link function; β_2-_ represents the immediate level change at pandemic onset; β_3-_ quantifies the slope change following that intervention; ^b^SE (Standard Error): standard error of the estimated β, indicating the precision of the coefficient estimate; ^c^RR (Rate Ratio): exponential of β (exp(β)), *model without seasonality and without lags in the autoregressive process;** model with seasonality and with 1 lag in the autoregressive process (AR(1)); ;*** model without seasonality and with 1 lag in the autoregressive process (AR(1)).

Source: Mortality Information System (SIM/SUS) | National Bureau of Statistics (IBGE)
